# Copyright Governance for Online Short Videos: Perspective of Transaction Cost Economics

**DOI:** 10.3389/fpsyg.2022.916670

**Published:** 2022-06-16

**Authors:** Mingxia Long

**Affiliations:** College of Literature and Journalism, Sichuan University, Chengdu, China

**Keywords:** online video, social media, platform governance, video piracy, cultural psychology

## Abstract

In recent years, copyright governance for short videos has become a hot issue of common concern in the academic community and the industry. Therefore, this study intends to explore the economic aspect of copyright governance in relation to the proliferation of infringing short videos. The short video industry of China has been taken as a case to demonstrate the copyright governance issue. Transaction cost theory has been applied to analyze the economic aspect of copyright governance in terms of four dimensions: bounded rationality, opportunism, environmental uncertainty, and asset specificity. From the perspective of transaction cost economics, the problem of short video infringement is observed to be essentially a market failure due to high transaction costs. In the short video market, substantial transaction costs are incurred in the legal transaction of copyright with these costs considered to be too high. This is especially the case when transaction costs exceed the net proceeds initially expected by short video users from the authorization, making it impossible to carry out the transaction and leading to infringement. To effectively control the copyright infringement of short videos, it is necessary to build a cross-platform information-sharing mechanism to reduce search costs, establish a unified copyright management platform to reduce coordination costs, and give full attention to the role of technical support to reduce regulatory costs.

## Introduction

Digitization and transnational communication networks bring new actors and interests into the sphere of copyright governance, generating new institutional dynamics due to the emergence of new technologies and market opportunities (Koutras and Papadopoulos, 2021). Notably, decision-making is shifting away from local settings to international settings. The fast advancement of information and communications technology (ICT) has resulted in a globally networked civilization increasingly reliant on massive amounts of global data exchange (Dusollier, 2020). However, owing to the substantial reliance on information connectivity, particular concerns crucial to the order and equity of information sharing, such as intellectual property rights, are being prioritized (Jiang, 2015). Copyrights, a key category of intellectual property rights that deal with the ownership of innovation, have become entangled in competing economic, political, and cultural interests (Engelbrekt, 2013). China has always been reluctant to develop a copyright regime, owing to the historically ingrained values of collectivity and Confucianism in Chinese culture, which have discouraged legal barriers between forms of artistic output (Cheng, 2019). As a result, widespread piracy has become a contentious aspect of Chinese culture, both in volume and influence, endangering political and economic stability locally and globally (Wang, 2017).

The popularity of the Internet and the rise of digital technologies have transformed former passive recipients of information into active content creators. A thriving participatory culture has increased public participation in creating short videos. According to the 48th statistical report “The Development of China’s Internet Network” released by the China Internet Network Information Center (CNNIC), as of June 2021, the scale of short video users in China has reached 880 million, 87.8% of the total netizens (Mingzhao, 2020). However, with this growth, the degree of infringement of short videos has also reached unprecedented levels. According to statistics, from January 2019 to May 2021, the 12,426 copyright monitoring centers monitored 30,095,200 suspected infringing short videos (Mingzhao, 2020). A short video is usually a new video format in which the film’s length is measured in seconds. It primarily relies on mobile smart terminals for quick shooting and editing. It may be shared and integrated smoothly into real-time social media networks. Short videos are not simply shortened versions of longer videos (Ji, 2021), as they also have solid social features, a low threshold for creation, and fragmentation, all of which are important in today’s fast-paced world (Maneeratana and Phongtongjalearn, 2019). The main two types of infringement in the short video market are (1) the infringement of original short video works and (2) the infringement of short videos against other copyrighted works (Li and Suping, 2020). The short video infringement issue therefore is of concern to the sustainable development of the short video industry and relates to the construction of a healthy competition system in the content industry market (ENSAfrica, 2010).

In recent years, copyright governance of short videos has become a hot issue of concern in the academic community (Wang, 2017). Most studies attribute the proliferation of infringing short videos to the copyright law seriously lagging behind the current reality of the development of network technology (Lintaman, 2020) and then discuss the governance of short video copyright from the legal science perspective (Tan, 2018). For example, Xiaojun and Yudi (2019) believed that the existing “notice–delete” rule can only achieve the purpose of copyright governance after the fact and cannot achieve systematic pre-event copyright governance. They add that setting the platform’s copyright filtering obligation is the key to copyright governance of short video platforms. As proposed by Chao and Ning (2021), the traditional behavior model should be broken, as it is a model in which the platform does not perform its obligation of active copyright review. The platform should conduct a prior review of whether copyright infringement has occurred in short video works, requiring them to bear the corresponding infringement liability according to the degree of subjective fault. As pointed out by Zhuliang (2020), self-media short videos have changed the ecological environment of the copyright system. To protect copyright owners, their passive licensing rights of “opting out” should be guaranteed. To protect the culture of public participation, if the work has a transformative use and does not affect the legitimate interests of the original work, this is regarded as fair use. In contrast to the above-mentioned view of managing short video infringement based on copyright protection, Shiquan (2021) believed that we should establish and improve the rules based on copyright sharing to fundamentally control short video infringement and its resultant chaos.

Although, in terms of academic theory (Halbert, 2019; Kaye and Gray, 2021) and judicial copyright practice (Li and Cheng, 2015; Cunningham and Craig, 2019; Caplan and Gillespie, 2020; Zhuliang, 2020), useful studies and explorations are available on the copyright governance of short videos, no consensus has been formed on how to deal with the problem of short video copyright infringement. Therefore, new perspectives need to be sought to decipher the governance dilemma posed by the proliferation of infringing short videos. Copyright essentially provides a market-based solution to the balance between incentivizing authors to invest in artistic works and promoting the dissemination of those works to the public (Dwivedi et al., 2021). In short videos, the issue of copyright governance belongs to both legal and economic categories (Cabrera Blázquez et al., 2018). This study applies the framework of transaction cost economics to analyze the logic of the occurrence of short video copyright infringement and then proposes an effective path to deal with this short-sighted infringement.

Considering the importance of the copyright governance for short videos and fulfilling the research gap, this study intends to address the research questions, “how copyright governance is working for short videos, and what are the economic consequences of the proliferation of infringing short videos?” To address the research questions, this study intends to explore the economic aspect of copyright governance in relation to the proliferation of infringing short videos.

The following is the format of our study. The second section deals with methodology. The third and fourth section provides findings and discussion section, respectively. After providing future study directions, we conclude our study.

## Methodology

To familiarize readers with the core theoretical and analytical framework, at first, we present a brief introduction to transaction cost economics. In the third section, we provide a comparative discussion based on transaction cost economics theory. Logical reasons are provided for the application of transactional costs and blockchain technology to start-up finance in the proposed theoretical model. Then, our model is compared to findings and arguments presented in the existing literature. In the fourth section, we provide recommendations to enhance copyright governance based on the study’s findings.

Transaction cost theory in economics is considered a theoretical tool with a wide range of uses. Economist Oliver Eaton Williamson (Williamson, 1998) pointed out that “[a]ny relationship, whether economic or other, as long as it manifests itself or can be expressed as a contracting problem, can be evaluated according to the concept of transaction cost economics.” Applying the transaction cost economics framework to analyze the phenomenon of short video infringement and its governance problems, we can find the root cause of short video infringement and its governance dilemma and provide new ideas for short video copyright governance.

### Transaction Cost Economics Theory

In the 1930s, the economist Coase (1937) first introduced the concept of transaction costs into economic analysis. He highlights that transaction costs are the costs of using the price mechanism, including finding transaction partners, searching for market information, negotiating, signing contracts, supervising performance, and resolving breach of contract disputes to complete market transactions. In Coase’s view, the trading process requires the transaction subject to invest time, energy, and even economic costs, with the price paid for completing the market exchange deemed the transaction cost. Williamson likens the role of transaction costs in economic activity to friction in physics and further expands transaction cost theory (Williamson, 1998), finding that the level of transaction costs is affected by the characteristics of the transaction itself. In the broadest sense, transaction costs include the cost of making an exchange of anything that does not occur directly in the materials production process. High transaction costs often make the trading entity loses the motivation to conduct transactions and hinders the smooth progress of trading activities (Boudreau et al., 2007).

**Figure 1:**
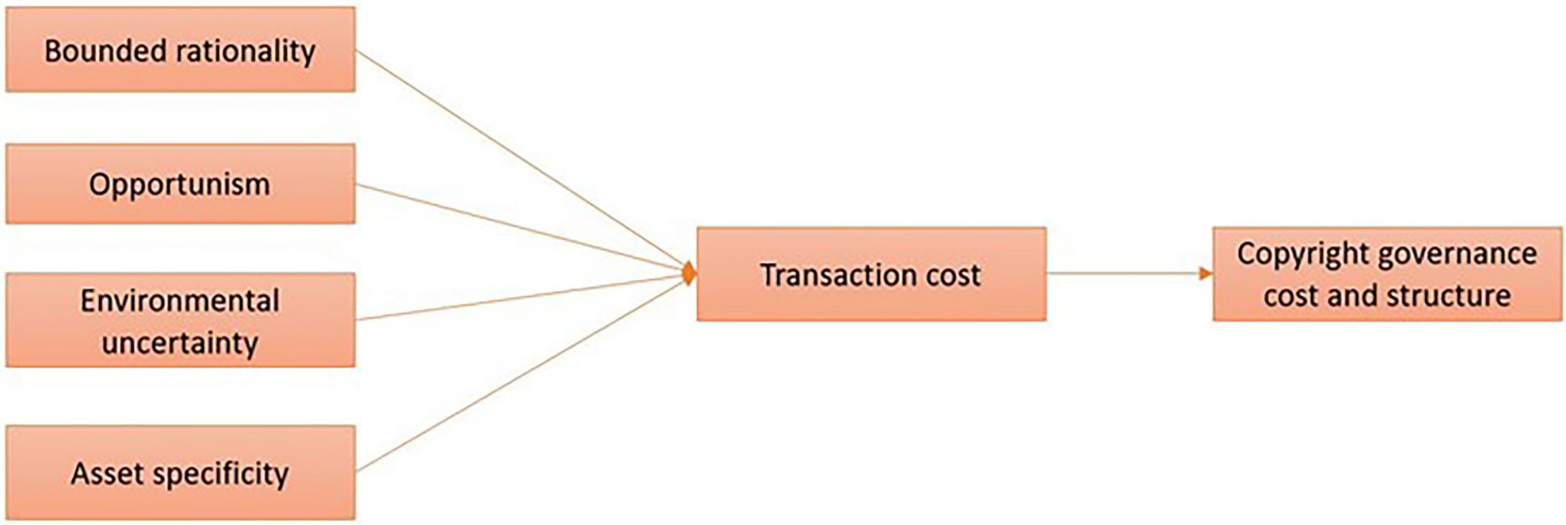
Analytical framework under transaction cost economics theory.

Since the publication of Williamson’s fundamental book *Market and Hierarchies*, transaction cost economics is considered one of the prominent theoretical models in the management and organization discipline (Mcclelland and O’Brien, 2011; Ahluwalia et al., 2020; Hoh and Tang, 2021). The theory attempts to explain the nature of the firm and predicts why certain activities are performed inside the firm, in contrast to the neoclassical economics view of the actions conducted by a free market system or a hybrid market arrangement, where parties to the agreement are interdependent in a nontrivial way (Lajili and Mahoney, 2006; Williamson, 2009). “Transactions” and “costs” are at the heart of transaction cost economics theory. The transaction transfers a unit of commodities or services, while the costs are the sum of the associated monetary and nonmonetary values involved in making the transfer possible (Zhuang et al., 2020). The major elements of the theory are bounded rationality, opportunism, environmental uncertainty, and asset specificity. The transaction cost economics model is depicted in Figure 1.

Bounded rationality reflects the attempt by transaction subjects to act rationally. However, due to limitations in subjects’ intelligence, knowledge, ability and other aspects, the complexity and uncertainty of the environment they face (i.e., environmental uncertainty), and the existence of asymmetric information, they can only achieve bounded rationality (Williamson, 2009). The second element is opportunistic behavior, that is, the transaction subject is motivated to seek its interests through improper means (Finkin, 2005). The act of evading economic responsibility occurs by internalizing the benefits and externalizing the costs in the nonequilibrium market (Carnahan et al., 2010). Asset specificity is another element that affects transaction costs (Boudreau et al., 2007). High asset specificity refers to an asset that cannot be easily redeployed for other purposes. In contrast, low asset specificity refers to an asset that may be used in other ways (Mcclelland and O’Brien, 2011). For instance, if a supplier agrees to make significant investments in assets that are applied to making a product for a certain buyer, the supplier has made the buyer’s assets unique to that buyer. If the supplier fails to meet the buyer’s needs, the supplier permits the buyer to pay less to avoid losing the significant value of specific assets.

In the short video market, copyright trading activities are complex (Li and Suping, 2020). It is difficult for transaction entities to eliminate both the limitations of bounded rationality and the tendency for opportunistic behavior, resulting in high transaction costs and frequent infringements.

### Analytical Context: Short Video Industry in China

Short video development is still in its infancy in China, although it began in 2011 in the United States (US). However, numerous domestic mobile short video social applications have shown remarkable potential, such as Toutiao’s Douyin and Tencent’s Meipai, as well as Pear Video, Ergeng, and other applications, which all have continually growing user bases. According to CBN Business Data’s “2017 Short Video Industry Big Data Insights,” the number of mobile video users in China reached 525 million in December 2017, with the number of short video industry users continuing to expand, resulting in a new explosion point for mobile video (Mingzhao, 2020).

As previously mentioned, the term “short video” means a video format in which the length of the film is measured in seconds rather than minutes. For quick shooting and editing, it mostly relies on mobile smart terminals, and it may be shared and seamlessly integrated in real time on social media platforms (Xiaojun and Yudi, 2019). Short videos are not simply shortened versions of longer videos, as they have powerful social features, a low threshold for creation, and fragmentation, all of which are important in today’s fast-paced world (Shiquan, 2021).

Take, for example, Douyin, a short video platform with a huge domestic user base and influence: while it uses the user-generated content (UGC) production approach, with users serving as both producers and communicators, it is simple to create and enter. Barriers to entry are minimal, and by using the UGC approach and being both easy to enter and create, Douyin is a great social product. Due to its users, Douyin is more than simply a place to watch and make short films, it is also a place to express yourself and share your life.

According to the “2017 China Short Video Industry Research Report,” a large number of mobile short video applications have been intensively launched since 2016, with Tudou transforming into a short video platform in April 2017; Toutiao Video being upgraded to Watermelon Video in June 2017; and second receiving renminbi (RMB) 100 million in financing in August 2017. The competitive pattern of the short video industry’s market has increasingly stabilized, and a steady commercial monetization model has emerged. In general, the short video market is evolving quickly and exhibiting a positive trend, resulting from the combined effect of the external environment and internal driving forces (Shiquan, 2021).

With regard to the external environment, the widespread availability of mobile smart terminals and the maturation of 4G mobile Internet technology provide critical technical support for short videos, while changes in user-content consumption demand have shifted the overall social environment from a graphics- and text-based era to a video-based era (Zhuliang, 2020). User-driven, advertiser-driven, and platform-driven are the three basic types of internal driving forces. The industry has progressed as a result of the large-scale increase in users. The value of short video marketing has attracted many advertisers, and numerous short video platforms have continued to emerge and develop in detail. As a result, the short video sector’s growth will undoubtedly become another factor for the growth of the mobile economy (Hare and Choi, 2019).

Furthermore, the rapid development of local short videos has resulted in the formation of a comprehensive production and distribution chain. Content-specific institutions, platform-specific institutions, and other types of institutions produce both content and platforms. Three mainstream media, namely, CCTV, People’s Daily, and Xinhua News Agency, represent content agencies; Toutiao, Tencent, Kuaishou, and Miaopai represent platform agencies. As a new communication style, the fragmented expression of short videos is convenient for producers and enjoyable for spectators. It contributes to the media industry by meeting people’s spiritual and entertainment demands, while giving the content of products and means of expression in news communication a fresh lease of life (Johnson, 2021). However, several issues have surfaced during the production of short videos, which should pique the interests of the industry and academia. If these issues are not resolved, their many undesirable societal consequences will have negative impact on the health and efficiency of China’s development.

## Results and Discussion

### Theoretical Model Analysis

Transaction cost theory is the key to understanding the frequent occurrence of short video copyright infringement. In line with transaction cost economics, transaction costs are believed to be a key factor affecting transactions, with high transaction costs hindering or even preventing the formation of the market. Similarly, in the short video market, the cost of a market transaction is higher than the transaction fee ordered by the court for infringement. The public will choose to abandon licensing negotiations or even ignore the issue of licensing and using the work (Ahluwalia et al., 2020). From the perspective of transaction cost economics, the issue of short video copyright infringement can essentially be seen as a market failure based on high transaction costs (Towse, 2005).

#### Bounded Rationality and Unintentional Infringements by the Transaction Subject

With the popularization of mobile intelligent terminal equipment, the simplification of video production technology, and the rise of various short video platforms, such as Vibrato and Kuaishou (Li and Suping, 2020), the traditional technical barriers to shooting, editing, and producing videos have been increasingly dissolved (Ji, 2021). Development of the Internet and digital technology has greatly reduced the marginal cost of copying, forwarding, sharing, and disseminating short videos, which have become a popular trend (Katzenbach, 2018). Everyone now can become the creator of short videos and the main trader of their copyright. In line with transaction cost economics, the transaction subject is believed to have limited knowledge, experience, and ability in concluding an actual transaction. The information collected and obtained are also limited, with it being impossible to know everything. When trading activities are simple, information are transparent and deterministic, and trading results are predictable, bounded rationality is not problematic. However, when bounded rationality is combined with uncertainty or complexity, this will greatly increase transaction costs as transaction subjects must expend more energy and time in collecting, organizing, and analyzing information to improve their rationality. In the short video market, no ordinary social media user can sufficiently understand copyright law to assess the legitimacy of their content generation activities. The user makes decisions based on limited rationality, resulting in many unconscious infringements.

The copyright of short videos has a considerable degree of complexity, which is concentrated in two aspects. First, the originality of short videos is ambiguous that directly affects whether the short video can become the object of copyright protection. China’s newly revised Copyright Law has adjusted the definition of “work” and introduced the concept of “audiovisual works” to include new content forms such as short videos in the scope of copyright protection of works (Zhang and Wu, 2019). However, this does not mean that all short videos can become the subject matter of copyright protection. According to the existing legal provisions, short videos must be original for them to have copyright (Hare and Choi, 2019). Compared to traditional film and television works, the length and limited space for expression of short videos have not led to consensus on the criteria for judging their originality. One view is that a minimum originality standard should be adopted: as long as the short video is independently created by the author and reflects a certain degree of personalized choice such as its arrangement, trade-off, and design, it should be protected by copyright (Aguiar et al., 2018). Another view is that short videos must reach a certain creative height and have a particular creative level before they can be identified as audiovisual works (Aguiar et al., 2018). China has not clearly defined and interpreted how to determine the originality of short videos at the legislative level. With ample space for discretion existing in judicial practice, it is difficult for users who share, forward, or use short videos to accurately determine whether the content materials they are using are protected by copyright (Ji, 2021).

#### Opportunism and Intentional Infringements by the Transaction Subject

Opportunism is an important behavioral feature of “economic subjects,” the term that refers to the nature of people in economic activities (Boudreau et al., 2007). Economic subjects will not hesitate to harm others and benefit themselves to maximize their protection and increase their interests. As a potential behavioral tendency, opportunism will be transformed into actual behavior when the conditions are in place, adversely affecting market transactions (Mcclelland and O’Brien, 2011). The uniqueness of the short video market facilitates opportunistic behavior in the use of copyright.

First, the complexity of the short video market can result in copyright infringement. These infringements do not necessarily lead to the loss of the commercial value of the original work. They may even maximize the copyright owner’s benefits as the public’s forwarding, sharing, secondary creation, and other acts provide an opportunity for the copyright owner to disseminate his/her work without additional marginal costs (Hoh and Tang, 2021). For example, the “2021 China Online Audiovisual Development Research Report” released by the China Online Audiovisual Program Service Association shows that 61.1% of users watch online video programs because they notice relevant highlights on platforms such as *Douyin* and *Kuaishou*. The popularity of short videos is usually short-lived, and strict copyright protections may make it difficult for creators to benefit from the first-mover advantage. This gives copyright owners an economic incentive to encourage the public to distribute and use their works without copyright restrictions (Lajili and Mahoney, 2006).

Second, the copyright owner will incur a high cost to protect his/her rights and may not be strongly proactive. Massive numbers of short videos are disseminated across various platforms *via* the network. Even if the work is infringed, addressing this is difficult for copyright owners (especially when the copyright belongs to individual creators; Kaye and Gray, 2021). Moreover, even if the copyright owner knows that his/her work’s copyright has been infringed, taking effective legal action is troublesome. The collection of evidence of short video copyright infringement and litigation are expensive and time-consuming, while many individual creators of the one work cannot face copyright infringement (Lintaman, 2020).

Third, as Internet service providers, short video platforms have insufficient motivation to review the copyright legitimacy of the content uploaded by users. In international practice, China has introduced the “safe harbor principle” in handling online infringement and does not require the platform party to bear the general obligation of active review. For instance, let us consider a case where the user has infringed and needs to notify the short video platform of the infringing content found by the copyright owner. After receiving the notice, the platform party shall promptly take measures such as deleting, blocking, and disconnecting the link (Wu et al., 2022). The uploading user shall bear the consequences of an infringement. Infringement is only constituted if the platform knew, or should have known, that the work provided by the user was infringing, and the link was not actively deleted or disconnected (Li and Cheng, 2015). In summary, infringement has a comparative advantage in transaction costs for short video users, promoting speculation.

#### Environmental Uncertainty

The underlying contradiction of information assets in the Internet context, especially copyrighted works, is due to their dual nature: They must produce money as economic objects, which means that unrestricted use, redistribution, and derivation of creative works must be prohibited (Boudreau et al., 2007). However, being creative objects, they must inevitably build on and inspire previous works, and thus, an endless flow of short videos must be permitted and fostered by legislation to sustain a continual creative process in society (Lajili and Mahoney, 2006). Copyrighted works, often known as information products, are generated for publication and distribution on the Internet’s highway of marketplaces. However, once short videos are posted online, they become common knowledge, freely available for anybody to use, duplicate, and change, whether for a charge or for no fee. As society is so interested in short videos, the legal architecture of copyright is built, guaranteeing writers, among other things, temporary sales rights and benefits. Crucially, in exchange for publishing their works, the producers of works are granted exclusivity like copyright (Ahluwalia et al., 2020).

Historically, the copyright of a work protects its holder’s creative works and most stereotyped outputs from illegal use. It is a legal right granted to the author, allowing the author to prohibit others from using their copyrighted work (Alén-Savikko, 2016). Referring to the right to “exclude others” from utilizing works protected as intellectual property, copyright infringement is characterized as “theft” of the copyright holder’s “property” that resides in their exclusive domain. The statement of property rights implies an exclusive possessory right over intangible bits of information. This privilege would empower the possessor to exclude the rest of the world from that work, which is viewed as purely informational (Johnson, 2021). At the same time, there is no question that a copyrighted work is more than simply a collection of data. The copyright system is a set of rules that control more than merely the management of digital information. Digitalization of copyrighted works unquestionably results in information aggregation, at the very least in the form of bits (Schovsbo, 2008). In the Internet context, where “information wants to be free,” it remains debatable whether information *per se* can correctly be regarded as property when a work undergoes this type of transition (Dusollier, 2020).

On the one hand, knowledge may be pirated for reasons of necessity. The correct information at the right time may completely transform an individual’s life. On the other hand, with information “wanting to be free,” the cost of its dissemination decreases. So now, these two aspects are battling it out. In contrast to limited and scarce material resources, intangible information included in copyrighted materials, such as short videos that become available online, is non-rivaled, which means that it is not diminished by its usage (Geiger and Jütte, 2021). Non-rivalry, according to experts, is the polar opposite of congestion. The pleasure of watching a football game, for example, is not diminished by the presence of a large number of viewers. In other words, when a creator’s work is made freely available online, the marginal cost of providing the work to an additional user is zero. As a result, consumption is needlessly rationed when an author or other rights holder charges for access to a work that becomes commercially available (Mcclelland and O’Brien, 2011). Users who refuse to pay the market rate are barred from using the work, even though they would have profited from it for free. As a result, social well-being is not maximized.

#### Asset Specificity and Video Infringement

Many short videos in the market recreate other copyrighted content materials (e.g., film and television drama works). Based on the Copyright Law’s principle of fair use of works, whether such secondary creation short videos constitute infringement needs to be analyzed according to various circumstances (Williamson, 2009). For example, if short videos are for personal study, research, or appreciation purposes, or if they are used to present, comment on a specific work, clarify a certain issue, or adequately refer to other published works (Boudreau et al., 2007), this may constitute fair use without the copyright owner’s consent, and the user may not need to pay remuneration. Specific and clear provisions on the criteria for appropriate citation do not exist in legislation, with judicial practice frequently considering elements such as the referenced work’s aim, character, and proportion to the whole work (Lajili and Mahoney, 2006). Another factor is if the citation has a detrimental effect on the original work’s usual use or the original work’s prospective market. Professional and complex judgments are required as to whether some adaptations constitute fair use, with this often beyond the capabilities of network users and short video platforms (Collins, 2014). Under the condition of bounded rationality, if a correct judgment is to be made on these issues, more time and energy must be devoted to understanding the relevant legal knowledge (Lipton and Tehranian, 2015). To avoid paying high transaction costs, users may prematurely stop searching for this knowledge, making their decisions based on limited knowledge and information, resulting in unintentional infringement when creating and disseminating short videos (Cunningham and Craig, 2019).

## Recommendations for the Governance of Short Video Infringement

Copyright transactions in the short video market are exchanges on works between rights holders and users. Transaction costs mainly come from search, coordination, and regulatory costs (Lintaman, 2020). High transaction costs will induce infringement by short video copyright users, especially when the transaction costs required for copyright transactions exceed the transaction surplus, and infringement has a comparative advantage in transaction costs compared to seeking authorization (Koutras and Papadopoulos, 2021). To effectively control the phenomenon of short video copyright infringement, the transaction cost of short video copyright needs to be reduced.

### Build a Cross-Platform Information-Sharing Mechanism to Reduce Search Costs

On completing any transaction behavior, a search process is undertaken between the two parties to the transaction, incurring the resulting search cost, which is the cost necessitated to obtain evidence and exchange information on the transaction object (Graef and Prüfer, 2021). For products for which the copyright is unknown, the user needs to pay the cost of searching for copyright information. If users need to spend too much time and effort to determine whether the content materials they are using are protected by copyright and to find a copyright owner, this may cause the transaction to fail (Shiquan, 2021). The complexity of short video copyright means that users seeking to have legal use of content materials are required to pay high transaction search costs, resulting in infringement by many users in the short video market (Cabrera Blázquez et al., 2018). Building a cross-platform information-sharing mechanism can allow users to easily and quickly obtain relevant information, which helps to prevent their unconscious infringement. At the same time, relatively transparent information can also reduce users’ opportunistic behavior, preventing intentional infringement (Wang, 2010).

The seeking of authorization by the users of short videos largely depends on the search costs required to find copyright information. The combined effort of government, industry associations, platforms and creators, and other short video-related stakeholders is needed to build a unified short video audiovisual copyright database that is free and open to the public (Su et al., 2018). This can help the public in determining whether the content materials they want to use are copyrighted works, while developing their understanding of the ownership of the work’s copyright and owners’ contact information, reducing the search cost before the transaction, and promoting the smooth progress of transaction activities. At the same time, even if short videos are copyrighted works, the purpose of short video creators needs to be considered. Some short video creators hope to obtain direct economic benefits through copyright licensing. In contrast, others widely disseminate their works through free sharing of copyright and then indirectly obtain economic benefits through traffic monetization (Graef and Prüfer, 2021). Clear labeling on short video works is an effective way for the public to quickly identify whether short video creators are willing to share copyright for free, that is, all short video works that are clearly marked as “may not be used without permission” need to be authorized to be disseminated or used and those that are not labeled are presumed to be free to use (Li and Suping, 2020).

### Establish a Unified Copyright Management Platform to Reduce Coordination Costs

The cost of coordination refers to the cost of making copyright trading decisions and executing trading activities. According to China’s Copyright Law, the dissemination and use of copyrighted short video works should follow the principle of “authorizing first, then using,” in addition to complying with the conditions of “fair use” and “statutory permission to use” (Geiger and Jütte, 2021). In the current short video market, whether it is the use of original short video works or the secondary creation of traditional film and television works, no clear copyright licensing channel exists, so it is difficult for users to efficiently and conveniently obtain the copyright owner’s authorization (Cabrera Blázquez et al., 2018). The number of works that user of original short video works can access is enormous. Individual negotiations, cooperation, and contracts with multiple small copyright owner entities are prohibitive in terms of time, energy, and economic costs (Dwivedi et al., 2021). For secondary creators of film and television programs, these programs often have many copyright holders, with it being a complicated, time-consuming, and expensive process to identify and contact each of them and negotiate the price to finally obtain a secondary creation license.

Moreover, many ordinary individual creators are unlikely to have access to the film party or the publicity team, making it even more challenging to obtain the copyright owner’s authorization (Wang et al., 2017). In the short video copyright market, the average transaction frequency between subjects is generally not high due to the instability of the transaction object. The “first authorized, then used” model is followed, which requires short video users to pay high coordination costs. This causes short video users to treat copyright authorization in a negative way, often disseminating or using the work in an infringing manner first and then dealing with it when the copyright owner claims infringement remedies (Su et al., 2018).

When copyright owners and users are highly dispersed, establishment of a unified copyright management platform will simplify the purchasing of licenses by introducing a fixed interface. This would reduce the cost of coordination, enabling the market to play a role and helping to promote the legal use of audiovisual works (Fan, 2019). A more applicable management method is to carry out centralized licensing management of short video copyright. What is needed is the establishment of industry-led, nonprofit short video copyright collective management organizations, such as the Short Video Copyright Collective Management Association, to represent users (including those using short videos and secondary creators of film and television works) to communicate with the copyright owner to obtain authorization. These organizations can also represent the copyright owner to collect statutory license fees from users. In this way, users do not have to face many rights holders. They would only need to negotiate the conditions for use of the work with the short video collective management organization and pay royalties *via* the organization (Wang et al., 2017). A unified copyright management platform can improve the efficiency of copyright transactions and reduce the cost of time, energy, and financial resources expended by users in negotiations with each copyright owner. This would also promote the formation of the industrial ecology of “first authorized, then used” for short video creation (Li and Suping, 2020).

### Role of Technical Support and Technological Development in Reducing Regulatory Costs

Timely monitoring, discovery, and investigation of infringement comprise the premise of effectively regulating the use of short video copyright and ensuring the smooth progress of short video copyright trading activities. However, this remains challenging to supervise in practice (Feng, 2017). On the one hand, it is very easy to produce short videos, with their low-cost production threshold level and substantial content production. For example, according to public data, as of June 2020, in the first quarter of 2020 alone, nearly 300 million users on Kuaishou published works, with 1.8 million content creators submitting 4.9 million video content on Station B (Li and Suping, 2020). Thus, it is difficult for rights holders to find infringed content in the massive amount of content. On the other hand, short videos spread rapidly, and infringing works are disseminated throughout the network or even on transnational platforms, making it complicated to check and confirm infringing content (Ji, 2021). The high cost of regulation results in inadequate regulation, which contributes to the proliferation of infringements.

With the rapid development of a new generation of information technology, such as blockchain, cloud computing, big data, and artificial intelligence (Fan, 2019), the application of these emerging technologies to short video copyright protection (Dwivedi et al., 2021) and the innovation of regulatory methods and means are conducive to reducing the difficulty of supervision, improving regulatory efficiency, reducing regulatory costs, and curbing infringement (Tan, 2018). For example, blockchain is a decentralized technology for protecting the security and privacy of online transactions (Ahluwalia et al., 2020). It also has the potential to be applied to copyright protection and management, including setting permissions to access and use online content, controlling licensing, managing distribution, tracking the source of piracy, and charging fees based on content usage (Wu et al., 2022). The development of cloud computing, big data, and artificial intelligence technologies provides broader possibilities for automation, intelligent detection, analysis, and processing of various types of infringing content, such as text, pictures, audio, and video (Su et al., 2018). The use of modern technical means to supervise infringing short videos has long been in place in other countries. For example, YouTube launched a Content ID system that compares the audio and video uploaded by users with the copyright content database and automatically filters the content with high matching to reduce infringement (Collins, 2014; Wary and Neelima, 2019). By 2017, 98% of copyright issues on YouTube were resolved through the Content ID system (Cheung, 2019).

Many large video platforms in China have also established different degrees and patterns of automated infringement detection systems (Zheng et al., 2020). The technical principle is similar to YouTube’s Content ID system that determines whether infringement is occurring by comparing uploading videos with copyrighted video content (Collins, 2014). For example, the video gene comparison technology independently developed by Sohu Video uses existing copyright videos, at a level numbering nearly one million, as the gene matrix (Mingzhao, 2020). A fingerprint file represents the unique identifier for the video by extracting the video color and advanced semantic features (Zheng et al., 2020). ByteDance’s self-developed Spiritual Awareness System generates a unique “content fingerprint” file for each uploaded video content and compares this “content fingerprint” with other videos uploaded to the platform (Zhang and Zhang, 2012). Technological development has made it feasible to quickly and efficiently identify and deal with infringement (Sun and Zhang, 2021). The development and application of short video copyright supervision systems to intercept, block, and filter relevant infringing videos can compensate for the shortcomings of systems that rely solely on manual supervision pressure, with their high cost and poor effect, and effectively prevent users from uploading and disseminating infringing content.

## Conclusion

With the rapid development of China’s short video industry, the problem of short video copyright infringement has become increasingly prominent. To some extent, copyright issues have become a stubborn disease affecting the development of the short video industry. Relevant legislative, judicial, and administrative departments are taking several measures to rectify the chaos of short video copyright infringement. For example, since 2018, the rectification of short video copyright has been regarded as the key task of the Sword Network’s special action. China’s newly revised Copyright Law has significantly increased the cost of infringement, as well as increasing the crackdown on infringement by introducing a punitive damages system with higher amounts for infringement damages.

Diverse methodologies with different theoretical bases can construct a complete framework for understanding the ordering of the short video industry and the circulation of short videos. Transaction cost economics and its economic and institutional theory variants are popular and informative methods. According to transaction cost economics, the short video piracy problem is simply a market failure caused by excessive transaction costs. The legal copyright transaction in the short video market entails significant transaction expenses. Transaction costs are high, particularly when they exceed the net revenues anticipated by short video consumers from the authorization, rendering the transaction difficult to complete and resulting in infringement. The key to solving the problem of market failure caused by transaction costs lies in reducing transaction costs and making transactions relatively easy, improving the realizability of trading motivation and the possibility of trading.

## Data Availability Statement

The original contributions presented in the study are included in the article/supplementary material, further inquiries can be directed to the corresponding author.

## Author Contributions

ML designed the research plan, collected the data, analyzed the data, wrote the manuscript, revised the manuscript, checked the final version of the manuscript, and approved it for publication.

## Conflict of Interest

The author declares that the research was conducted in the absence of any commercial or financial relationships that could be construed as a potential conflict of interest.

## Publisher’s Note

All claims expressed in this article are solely those of the authors and do not necessarily represent those of their affiliated organizations, or those of the publisher, the editors and the reviewers. Any product that may be evaluated in this article, or claim that may be made by its manufacturer, is not guaranteed or endorsed by the publisher.
